# Exploiting epigenetics for the treatment of inborn errors of metabolism

**DOI:** 10.1002/jimd.12093

**Published:** 2019-04-22

**Authors:** Martijn G. S. Rutten, Marianne G. Rots, Maaike H. Oosterveer

**Affiliations:** ^1^ Department of Pediatrics University of Groningen, University Medical Center Groningen Groningen The Netherlands; ^2^ Department of Pathology and Medical Biology University of Groningen, University Medical Center Groningen Groningen The Netherlands

**Keywords:** DNA methylation, (epi)genome editing, gene correction, histone modifications, inherited metabolic disease, therapy development

## Abstract

Gene therapy is currently considered as the optimal treatment for inborn errors of metabolism (IEMs), as it aims to permanently compensate for the primary genetic defect. However, emerging gene editing approaches such as CRISPR‐Cas9, in which the DNA of the host organism is edited at a precise location, may have outperforming therapeutic potential. Gene editing strategies aim to correct the actual genetic mutation, while circumventing issues associated with conventional compensation gene therapy. Such strategies can also be repurposed to normalize gene expression changes that occur secondary to the genetic defect. Moreover, besides the genetic causes of IEMs, it is increasingly recognized that their clinical phenotypes are associated with epigenetic changes. Because epigenetic alterations are principally reversible, this may offer new opportunities for treatment of IEM patients. Here, we present an overview of the promises of epigenetics in eventually treating IEMs. We discuss the concepts of gene and epigenetic editing, and the advantages and disadvantages of current and upcoming gene‐based therapies for treatment of IEMs.

1

Compensating the primary genetic defect is currently considered as the ultimate treatment strategy for inborn errors of metabolism (IEMs). In conventional gene therapy, a vector containing the correct coding DNA (cDNA) sequence of the defective gene is delivered into the host.^1^ This method actually represents a compensation approach, as the endogenous genetic defect is not corrected. Gene therapy has been successfully applied in preclinical models for IEMs, including glycogen storage disease type 1a (GSD Ia; OMIM #232200), familial hypercholesterolemia (OMIM #143890), hemophilia B (OMIM #306900), ornithine transcarbamylase (OTC; EC 2.1.3.3) deficiency (OMIM #311250), hereditary tyrosinemia type 1 (HT1; OMIM #276700), and alpha‐1 antitrypsin deficiency (OMIM #613490).^2^, ^3^, ^4^ As a consequence of these successes, various gene therapy trials are currently ongoing. For example, recently, a human phase 1/2 trial has started in adult GSD Ia subjects (NCT03517085). The ultimate goal of such viral gene therapy is to restore production of the functional protein for sustained periods. For those IEMs in which the corrected proteins should be either secreted by or expressed in easily transduced cells (eg, hepatocytes),^5^ gene therapy would indeed provide a realistic cure if the transgene insertion could be targeted to harmless sites in the genome. However, when using conventional viruses, the lack of integration control may cause insertional mutagenesis and consequently severe side effects such as cancer.

Next to these safety considerations, conventional compensatory gene therapy has several other disadvantages. First, the size limitation of cDNA that can be cloned into a viral vector may compromise viral therapy for genes with a long coding sequence. Second, lack of control over expression of the newly introduced cDNA might compromise therapy effectiveness. Third, the need for multiple vectors to express different isoforms of the gene (or the selection of one specific cDNA isoform for transfer) might compromise gene therapy for certain diseases. For example, when a mutation affects multiple isoforms of the same protein with tissue‐selective expression, as is the case in hyperammonemia (OMIM #6126652 / #219150),^6^ a combination of viral vectors would be needed to correct the disease. Thus, although compensation gene therapy can provide an actual cure for IEMs, its disadvantages urge the exploration of alternative therapeutic opportunities.

Gene editing refers to modification of the DNA sequence at a precise genomic location. This approach involves different DNA targeting techniques such as meganucleases (MNs), zinc finger nucleases (ZFNs), transcription activator like effector nucleases (TALENs), and the currently widely used clustered regularly interspaced short palindromic repeats (CRISPR)‐Cas9 system, which all alter the genome of the host organism at the locus of choice^7^, ^8^, ^9^ (Figure 1). MNs are endodeoxyribonucleases, proteins with the capacity to recognize a 12‐40 base pair double‐stranded DNA‐sequence and cut DNA. ZFNs and TALENs both consist of transcription factor‐based DNA‐binding domains fused to a nuclease. With ZFNs, each zinc finger protein (ZFP) recognizes a three base pair‐DNA sequence. In the case of TALENs, each TAL protein consists of 34 amino acids, with amino acids 12 and 13 determining the recognition of one specific DNA base pair. By using multiple ZFNs or TALENs, a longer DNA sequence can be targeted, thereby increasing specificity. In contrast to MNs, ZFNs, or TALENs, CRISPR‐Cas9 uses a RNA‐based DNA‐binding strategy. The CRISPR‐Cas9 system consists of a single‐guide RNA (sgRNA) and the Cas9 protein. The sgRNA is a combination of CRISPR RNA(s) (crRNA[s]) and a trans‐activating crRNA of the original class II CRISPR‐Cas9 system, including a ~20 nucleotide RNA sequence that can be designed complementary to a specific location in the genome. The sgRNA guides the Cas9 nuclease to virtually any desired genomic location, after which Cas9 creates a DNA double‐strand break (DSB) at this targeted site.

**Figure 1:**
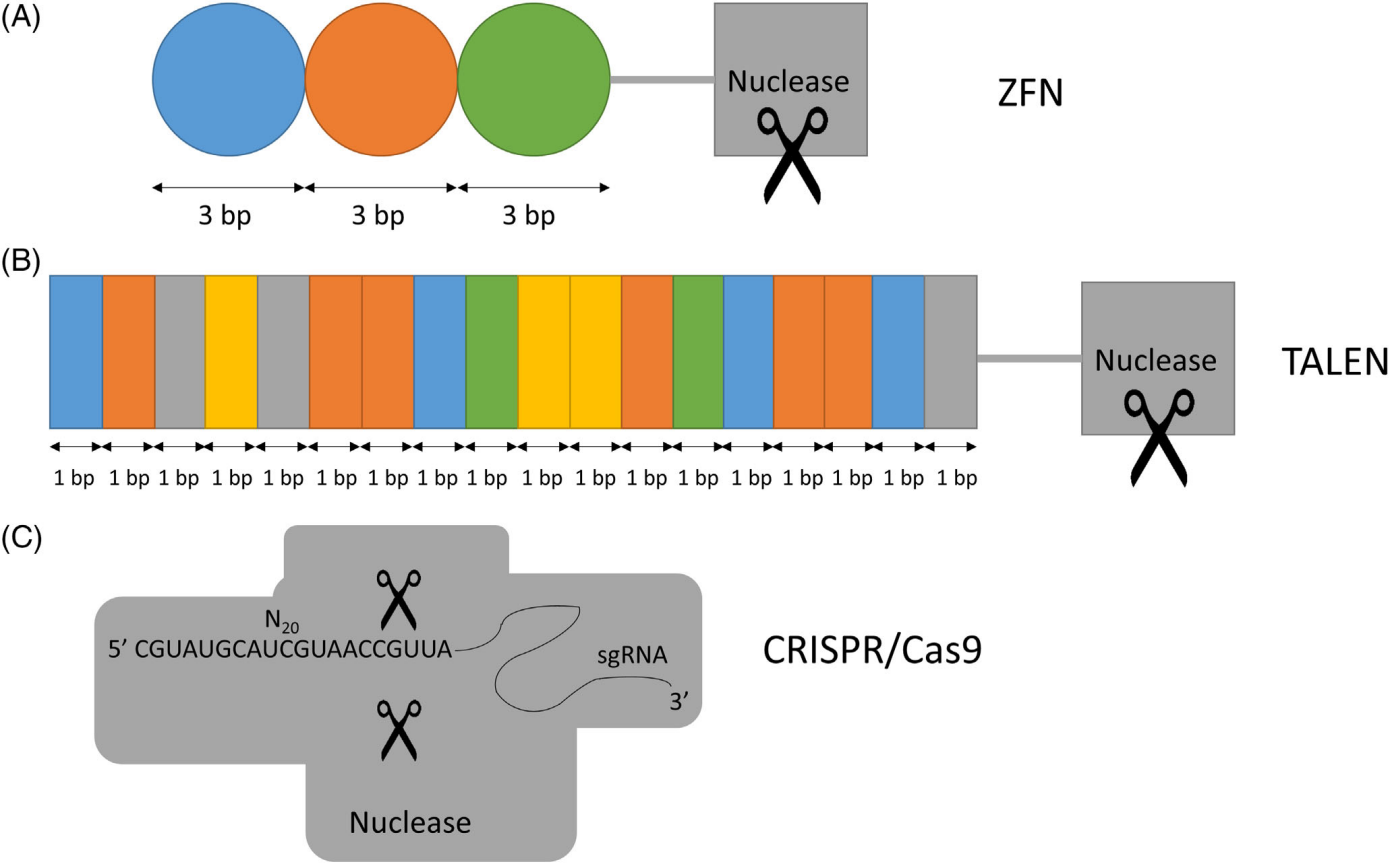
Schematic overview of the three main gene editing tools. A, Zinc finger nuclease (ZFN), consisting of a DNA‐cutting nuclease domain (gray box), and a protein‐based DNA‐binding domain of three zinc finger proteins (colored circles), each recognizing a three base pairs (bp) DNA sequence. Hence, this ZFN recognizes a 9 bp genomic sequence. B, Transcription‐activator like effector nuclease (TALEN), consisting of a DNA‐cutting nuclease domain (gray box), and a protein‐based DNA‐binding domain of 18 TAL effector repeats. Each TAL effector consists of 34 amino acids, typically highly conserved, with positions 12 and 13 being variable and determining the specific recognition of one DNA bp. Hence, this TALEN recognizes a 18 bp genomic sequence. C, Clustered regularly interspaced short palindromic repeats (CRISPR)‐Cas9 system, consisting of a DNA‐cutting nuclease (gray box), with two sites of nuclease activity, and a RNA‐based DNA‐binding domain consisting of a single guide RNA (sgRNA), with the variable 20 nucleotide RNA‐sequence determining recognition of a 20 bp complementary genomic sequence

Gene editing–induced DSBs are subsequently repaired by either non‐homologous end joining, usually resulting in inserts or deletions (indels) causing mutations and often a dysfunctional protein, or by homology directed repair (HDR). When a DNA repair template is present, HDR can insert (part of) a gene, or correct a mutated gene. Especially this latter option is of particular interest for treating IEMs because it enables the endogenous correction of the genetic defect of the IEMs in a one‐and‐done approach. This overcomes several downsides of gene therapy: it repairs the gene mutation at the endogenous site, rather than providing the cell with the entire cDNA sequence of the correct gene in a random location; the expression of the gene is under its endogenous control, as opposed to uncontrollable cDNA expression with conventional gene therapy; and finally, the correction of the mutation is expected to result in expression of all potential isoforms of the gene. The recently introduced CRISPR‐Cas9 technique is currently more widely used than ZFNs or TALENs because of the distinction between the DNA‐recognition domain (sgRNA) and the nuclease domain (Cas9), which makes it time‐ and cost‐effective to use. Targeting other genes only requires design of additional sgRNAs without the need to engineer different nuclease fusions. To date, CRISPR‐Cas9 has already been successfully used in various in vitro studies and in vivo animal models. In preclinical models for IEMs including OTC deficiency, HT1, mucopolysaccharidosis type II (MPS II; OMIM #309900), and GSD Ia,^3^, ^10^, ^11^, ^12^, ^13^ ZFNs and CRISPR‐Cas9 techniques have been used to correct the mutated gene or insert the correct cDNA. These techniques have progressed to clinical trials, for instance to treat lung cancer patients (OMIM #211980) by administration of their own T cells after these were isolated and genetically modified ex vivo.^14^ Recently, the first test of in‐body (in vivo) ZFN gene editing in humans has shown encouraging results by inserting a healthy copy of the iduronate‐2‐sulfatase (IDS, EC 3.1.6.13) gene into liver cells to treat MPS II.^15^

The main advantage of gene editing techniques is evident as IEMs can be corrected, rather than compensated, at the root: the actual genetic defect. In case the corrected protein is secreted, the genetic modification only needs to take place in a limited amount of cells. However, gene editing techniques also pose several important downsides that currently limit their clinical potential. Most importantly, gene editing may induce a p53‐mediated immune response^16^ and potential off‐target effects.^17^ Although it is currently difficult to accurately and efficiently predict the frequency and severity of off‐target effects, also because chromatin environments are different in different cell types,^18^ specificity of gene editing remains critical considering that DNA alterations are maintained life‐long. Especially when off‐target effects occur in the germline, transmission to offspring may result in unforeseen detrimental consequences.^19^ As a consequence, gene editing tools such as CRISPR‐Cas9 are a frequent topic of (moral) discussion, particularly after the recent birth of the first two human genome‐edited babies.^20^

Thus, although correction of the primary defects in IEMs would be preferred, the downsides of the available gene editing techniques stress the need for alternative therapeutic approaches. In this respect, it should be emphasized that IEM pathophysiologies are generally not solely related to the primary genetic defects, but also to the subsequent adaptations by the patient's cells and organs. This is exemplified by the lack of genotype‐phenotype correlations in certain IEMs,^21^, ^22^, ^23^, ^24^, ^25^, ^26^ illustrating the relevance of alternative mechanisms, such as epigenetics, to explain clinical heterogeneity between patients.^27^, ^28^ Thus, the distinction between the primary genetic defect and the subsequent adaptations is important, as it may allow for different, yet complementary, effective therapeutic approaches that aim beyond the primary defect. To distinguish between these two aspects, we refer to altered intracellular/extracellular metabolite levels, aberrant cellular signaling, or gene expression dysregulation^29^ as the secondary adaptations. There are multiple ways to interfere with secondary adaptations, for instance by targeting the intracellular and extracellular levels of metabolites and cofactors, by restoring aberrant cellular signal transduction pathways, and/or by means of gene expression regulation. Treatment of IEMs via reversal of the downstream effects of a genetic mutation can be achieved through correction of aberrant expression levels of disease‐associated genes. In this respect, gene editing tools can be used to modify gene expression levels by inactivating the DNA nuclease domain. One way to repurpose gene targeting tools is through fusion of epigenetic effector domains to the nuclease‐inactivated DNA‐binding platforms to modulate epigenetic signatures.

Epigenetics refers to the study of heritable changes in the phenotype that do not involve changes in the DNA sequence, but concerns alterations in DNA methylation and modifications of histones, hence affecting gene expression. For example, DNA methylation of a gene promoter generally leads to lower expression of that gene. Histone acetylation generally results in a loosened chromatin structure and thus increased gene transcription, while histone methylation can have both repressing or activating roles based on the amino acid of the histone that is modified. Besides acetylation and methylation, multiple other forms of histone modifications exist, including phosphorylation, SUMOylation, ubiquitination, and, perhaps more relevant for IEMs, citrullination, ADP‐ribosylation, O‐glycosylation, succinylation, and malonylation.^30^ In addition, metabolic activities are directly linked to DNA and histone modifications.^31^ For example, acetyl‐coA is a central metabolite that also serves as co‐substrate in histone acetylation, levels of nicotinamide adenine dinucleotide (NAD^+^) affect sirtuin‐dependent histone deacetylation, S‐adenosyl‐methionine and S‐adenosyl‐homocysteine regulate histone and DNA methylation, and mitochondrial oxidative metabolism is closely linked to histone demethylation.^31^ Considering this association between DNA/histone modifications and metabolism, epigenetics might provide a link between genetic defects and clinical phenotypes and may offer novel opportunities for refined diagnostics and treatments of IEMs with great potential. Although still a nascent field, the importance of epigenetics in IEMs is increasingly recognized, as exemplified by a review on the role of epigenetics in lysosomal storage disorders.^27^ Altogether, IEMs are associated with epigenetic changes, and epigenetics represents an interesting novel field for the treatment of IEMs.

Because epigenetic changes influence gene expression regulation, they can be targeted to correct or compensate aberrant gene expression in IEMs, with similar outcomes as gene therapy and gene editing. Several epigenetic drugs (“epi‐drugs”) have already been FDA approved, and in the oncology field numerous clinical trials with such compounds are currently ongoing,^32^ reflecting the promising therapeutic potential of targeting epigenetics. The main disadvantage of epi‐drugs, however, is the fact that they act genome‐wide rather than in a gene‐specific manner, increasing potential unwanted side effects and thereby limiting wide clinical applications. A technique that appears to be very suitable for safe, yet stable, therapeutic modulation of gene expression (as well as for compensation/alleviation of genetic mutations) is epigenetic editing (EGE,^33^, ^34^, ^35^). By combining epigenetic enzymes with the advantages of DNA‐targeting techniques, EGE directly exploits the reversibility of epigenetic changes in a gene‐specific manner. EGE is based on the programmable DNA‐binding platforms (ZF proteins [ZFPs], TALEs, or CRISPR‐Cas9), where the nuclease activity is removed or deactivated, thereby preventing manipulations of the DNA sequence. Rather, the DNA‐binding protein (ZF/TALE) or the deactivated nuclease (“dead” Cas9, or dCas9, in case of CRISPR‐[d]Cas9) serves as a “shuttle” protein to which other proteins can be fused, including artificial transcriptional activators, repressors, or other (co)factors involved in expression regulation, that allow transient gene expression modulation (Figure 2). In the case of EGE, epigenetic enzymes are fused to the deactivated nuclease to reprogram epigenetic signatures. These enzymes can be epigenetic writers, such as DNA methyltransferases or histone lysine‐ or methyltransferases, or epigenetic erasers, such as enzymes involved in removing DNA methylation or histone deacetylases/demethylases. EGE thus provides an innovative approach to not only study chromatin biology and causality in gene expression regulation, but also to ultimately potentially be used as a therapeutic tool.

**Figure 2:**
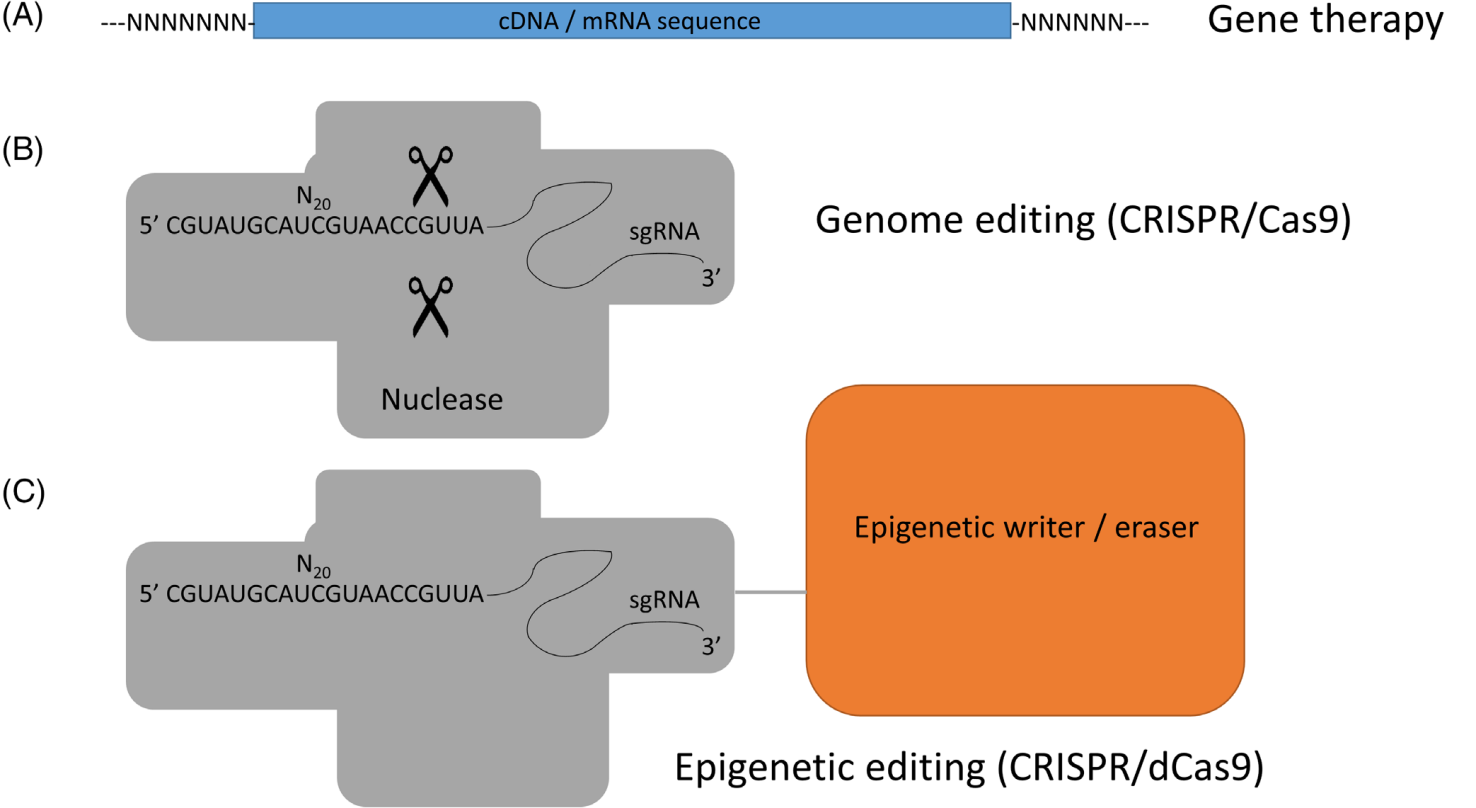
Comparison of different strategies to treat IEMs. A, Conventional gene therapy, based on the introduction of a cDNA or mRNA sequence encoding a correct version of the mutated gene. As a result, the defect is compensated without altering the genomic sequence of the host. B, Gene editing using (in this case) CRISPR‐Cas9, which aims to correct the mutated gene by altering the genomic sequence of the host at a precise location, or which can be used to modify the expression of proteins that compensate for the genetic defect via alterations in the genome. C, Epigenetic editing using an epigenetic writer or erasers fused to (in this case) the CRISPR‐dCas9 system to compensate for the genetic defect, for example, by increasing the residual expression of a mutated protein to enhance its activity, or by modifying the expression of proteins capable of compensating for the genetic defect, via gene‐specific alterations in the epigenetic landscape

EGE overcomes several downsides of the other approaches discussed earlier. Gene expression modulation via EGE, for example with a single sgRNA and an epigenetic enzyme fused to dCas9, achieves a natural, endogenous expression control, more closely mimicking nature than cDNA overexpression, inducing/targeting all isoforms, and without issues related to size. In some cases, epigenetic changes are mitotically stable,^36^, ^37^ implying a sustained therapeutic effect, and rendering the technique of EGE a one‐and‐done approach, similar to gene editing. Although similar to gene editing tools EGE might suffer from off‐target effects, the reversible nature of epigenetic changes allows for relative easy correction of these unwanted effects. So, while on one hand epigenetic changes can be actively overwritten by EGE, these changes can subsequently be copied during cell divisions, allowing stable effects. Indeed, during differentiation of cells, epigenetic signatures are rewritten to stably program the cell type‐specific gene expression profiles. These epigenetic signatures are subsequently copied to daughter cells during mitosis, ensuring the maintenance of cell identity. Despite this epigenetic memory, cells can adapt their epigenetic signatures in response to prolonged environmental changes. This adaptation of cells indicates the dynamics of epigenetics and is reflected in a common definition of epigenetics: heritable, yet reversible, changes in gene expression, not encoded in the DNA sequence. In EGE, the forced presence of an epigenetic writer or eraser at a genomic locus changes the epigenetic signature. Such changes can be copied/maintained by the epigenetic maintenance enzymes, allowing stability of the newly introduced mark. Given this characteristic of epigenetic changes, EGE is generally considered safer than gene editing, as no genetic changes are introduced, but might be equally effective, as stable expression modulation can be achieved.^36^, ^37^, ^38^

There are several examples of successful application of EGE using ZFs and CRISPR‐dCas9 in preclinical models. Research has shown that EGE can be applied in a gene‐specific manner for DNA (de)methylation and histone modifications, and that these changes might translate into physiological changes.^37^, ^39^, ^40^, ^41^, ^42^, ^43^, ^44^, ^45^, ^46^ EGE is also potentially interesting for the treatment of IEMs. For example, reducing *PCSK9* in familial hypercholesterolemia expression may reduce plasma cholesterol levels.^3^ In addition, EGE can also be applied in IEMs to increase the expression of a mutated protein with residual activity. In case of complete loss‐of‐function mutations, EGE may be instrumental to increase the expression of proteins that compensate for the defect. Examples of IEMs where this strategy may be powerful include hereditary fructose intolerance (OMIM #229600), in which the isozyme aldolase A (EC 4.1.2.13) might be upregulated to correct for aldolase B enzyme deficiency,^47^ and GSD Ia, where the lysosomal α‐glucosidase (EC 3.2.1.20 and EC 3.2.1.33) could be increased to enhance glucose‐6‐phosphatase (EC 3.1.3.9) (G6Pase)‐independent glucose production.^48^ Thus, the fact that epigenetic changes play a role in IEMs^27^, are reversible, and may arise later in life as a consequence of the IEM and could potentially contribute to long‐term complications of the disease, makes EGE also an interesting tool to prevent or compensate for these complications, such as the development of cancer in IEM patients.^29^

Each approach discussed in this review has its specific advantages and disadvantages (Table 1). One of the major challenges of all methods involves the efficient delivery of the therapy's components. Delivery can be accomplished in different ways, for example, by using plasmids, adenovirus, retrovirus, lentivirus, adeno‐associated virus, or ribonuclear methods. Although an extensive discussion of this issue is beyond the scope of this review, it is important to stress that therapy delivery is expected to further improve in the near future.^1^ Related to the issue of efficient delivery is the risk for an immune response to gene therapy and gene/epigenetic editing, which further challenges delivery efficiency and sustained therapeutic effectiveness. Ongoing and future research will elucidate if sufficient progress can be made to circumvent this issue.

**Table 1:** Overview of the advantages and disadvantages of different gene‐based therapies

	Gene therapy (eg, cDNA)	Gene editing (eg, CRISPR‐Cas9)	Epigenetic editing (eg, CRISPR‐dCas9)
Advantages	Functional gene copy	Corrects primary defect	Enhanced residual activity
			Functional compensation
		Functional compensation	Natural, endogenous expression control
		Endogenous manipulation	Targets all isoforms
		One‐and‐done approach	Potential one‐and‐done approach
			Reversible
Disadvantages	Limited cDNA size	Off‐target effects	Primary defect not corrected
	Limited effectiveness	Irreversible	Off‐target effects
	Uncontrollable expression	Inadequate delivery	Inadequate delivery
	Uncontrollable insertion	Immune response	Immune response
	Multiple vectors for multiple isoforms		
	Inadequate delivery		
	Immune response		

In conclusion, while conventional compensation gene therapy using cDNA is currently the preferred treatment for IEMs, there are several challenges that may hamper its clinical application. Corrective gene editing circumvents many of the issues, as it repairs the genetic defect permanently, overcoming the need for the introduction of a full‐length exogenous cDNA sequence. However, the main disadvantage of gene editing is the risk for off‐target effects with irreversible alterations in the genomic sequence, which can become particularly harmful when occurring in the germline. EGE represents a less invasive, yet potentially stable therapeutic approach. It allows for reversible alterations in DNA methylation and histone modifications in a gene‐specific manner, hence reprogramming gene expression profiles in IEMs with clear advantages over conventional gene therapy and gene editing. Compared with gene editing, EGE is considered safe because it does not lead to alterations in the genome, but to modifications in the epigenome. Because such modifications are in principle reversible, correction of potential off‐target effects seems more feasible. Altogether, epigenetics is a relatively unexplored field in the pathophysiology of IEMs, while metabolic activity is directly related to epigenetic enzyme activity and epigenetic marks on DNA and histones. EGE has clear advantages over gene therapy or gene editing approaches and thus provides a potentially valuable and novel approach to treat IEMs.

## CONFLICT OF INTEREST

The authors declare no potential conflict of interest.

## AUTHOR CONTRIBUTIONS

M.G.S.R. contributed to writing the manuscript. M.G.R. and M.H.O. contributed to writing and reviewing the manuscript.
